# Does the Use of Antidepressants Accelerate the Disease Progress in Creutzfeldt–Jakob Disease Patients With Depression? A Case Report and A Systematic Review

**DOI:** 10.3389/fpsyt.2019.00297

**Published:** 2019-05-03

**Authors:** Yifan Liang, Yan Li, Huibin Wang, Xi Cheng, Meiting Guan, Shanshan Zhong, Chuansheng Zhao

**Affiliations:** Department of Neurology, The First Hospital of China Medical University, Shenyang, China

**Keywords:** depression, Creutzfeldt–Jakob disease, antidepressant, neurotransmitters, sleep wake disorders

## Abstract

**Background:** Creutzfeldt–Jakob disease (CJD) is a fatal neurodegenerative disorder characterized by rapidly progressive dementia. Growing evidence suggests that antidepressant usage was associated with dementia. Given the commonality of depression in CJD, it is necessary to investigate the effect of antidepressants on CJD.

**Methods:** First, we report a case of sporadic CJD (sCJD) with depression where the condition worsened rapidly after using a serotonin and noradrenaline reuptake inhibitor (SNRI) antidepressant. Second, a systematic literature survey was conducted to investigate the effect of antidepressants on the survival time of sCJD patients with depression. Thirteen cases plus our case were included for qualitative analysis. Twelve subjects were included in the Kaplan–Meier survival and Cox regression analysis. Finally, we provide a postulation of pathophysiological mechanism in CJD.

**Results:** The median survival time of all patients was 6.0 months, of which patients with SNRIs were significantly shorter than those with first-generation antidepressants (2.0 vs. 6.0 months; log rank, *P* = .008) and relatively shorter than those with nonselective serotonin reuptake inhibitors (SSRIs; 4.0 vs. 6.0 months; log rank, *P* = .090). In comparison with first-generation antidepressants, the use of SNRIs [hazard ratio (HR), 23.028; 95% confidence interval (CI), 1.401 to 378.461; *P* = .028] remained independently associated with shorter survival time.

**Conclusions:** The use of antidepressants, especially SNRIs, was associated with a shorter survival time of sCJD patients. The possible changes in neurotransmitters should be emphasized. Scientifically, this study may provide insights into the mechanism of CJD. Clinically, it may contribute to the early diagnosis of CJD.

## Introduction

Depression is common in the elderly. Its prevalence rate is as high as 11.19%, and this increases progressively with worsening cognitive impairment (1). The presence of depression is an acknowledged risk factor for dementia (2); it can even double the risk for Alzheimer’s disease (AD) (3, 4). Many reasons lie behind the prescription of antidepressant drugs, which increased dramatically from 1999 to 2014 (5). However, some studies have questioned whether antidepressants confer any benefits in terms of cognitive decline (6–9). Recently, a meta-analysis indicated that antidepressant usage was associated with AD/dementia (10). Tricyclic antidepressants (TCA) may be associated with a reduced risk (11) or no risk of dementia (12) for depressed patients, whereas nonselective serotonin reuptake inhibitors (SSRIs) antidepressant drugs, including monoamine oxidase (MAO) inhibitors, and serotonin and noradrenaline reuptake inhibitors (SNRIs) have been reported to possess an intermediate risk (11–13).

Creutzfeldt–Jakob disease (CJD), a fatal neurodegenerative disorder characterized by rapidly progressive dementia, is divided into the sporadic (sCJD), familial (fCJD), variant (vCJD), and iatrogenic (iCJD) subtypes (14). sCJD accounts for the majority, i.e., 85% of all CJD cases, with an annual worldwide incidence of one to two cases/million population (15). Although rare, the overall mortality rate of sCJD has been increasing since 1993 (16). There is no effective treatment for CJD, so it is important to identify modifiable risk factors for CJD and to delay disease progression. Psychiatric manifestations are often the first symptoms to appear in vCJD. Recent studies have shown that they are also more prevalent in sCJD than previously thought. Most commonly, these are exhibited as depression in 16%–37% of cases (17–19). While treatment of the psychiatric symptoms with hypnotics, anxiolytics, or antipsychotic drugs may offer relief to CJD patients, it appears that antidepressant drugs are ineffective (19). As is the case in some other dementias, the implication is that antidepressants may also fail to benefit CJD patients.

Here, we report a case of sCJD in which depression was the first symptom, and the condition worsened rapidly after the administration of an SNRI antidepressant. Subsequently, a systematic review of the literature was undertaken to explore the characteristics of sCJD patients with depressive symptoms as well as the effect of treatment with antidepressants on sCJD. This report aims to provide novel insights into the underlying causes and treatment of CJD and dementia.

## Case Report

**Figure 1:**
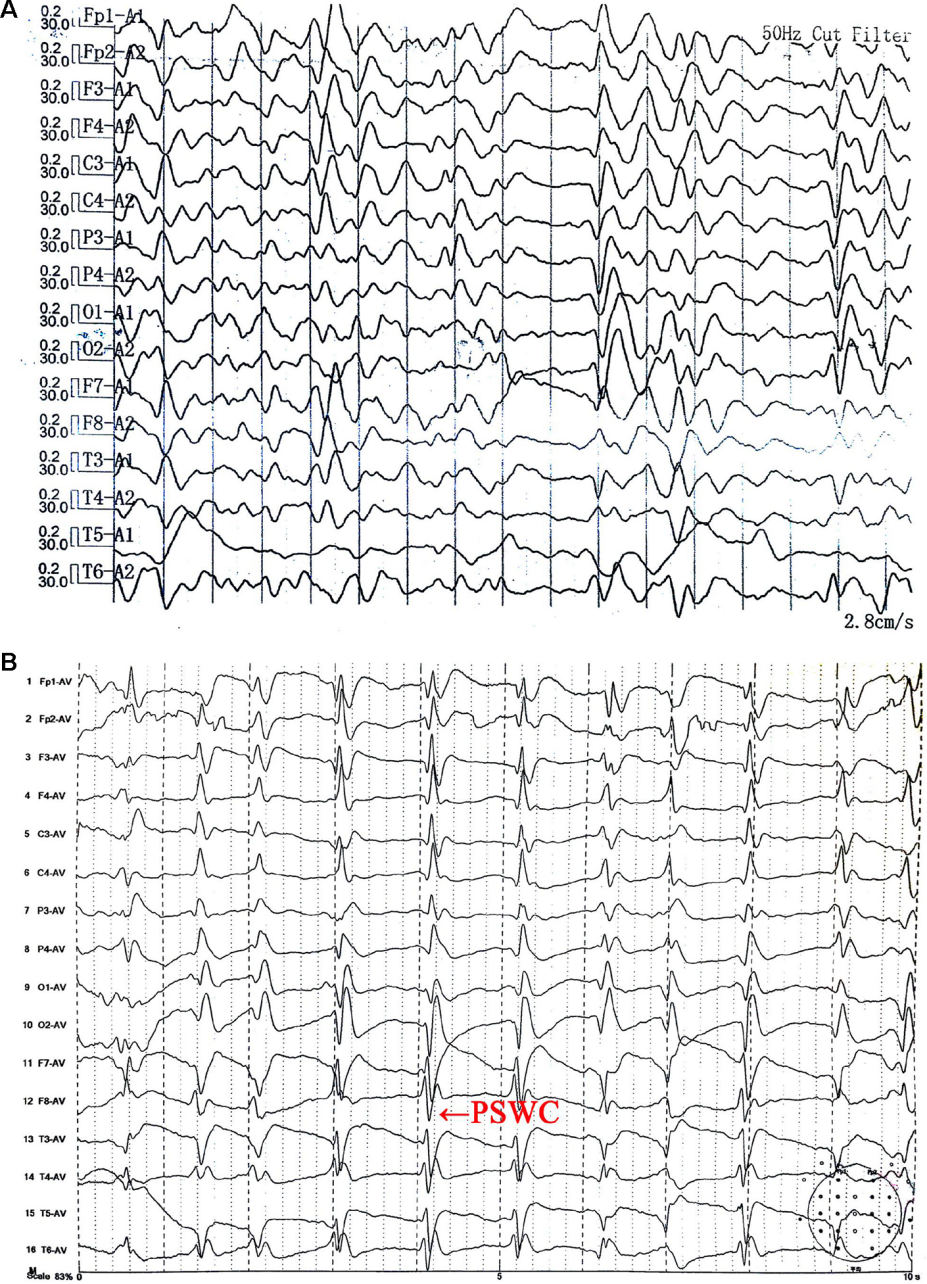
Electroencephalogram (EEG) **(A)** on October 26, 2017 showed generalized slow activity. The second EEG **(B)** on November 2, 2017 showed partially periodic sharp wave complexes (PSWC).

**Figure 2:**
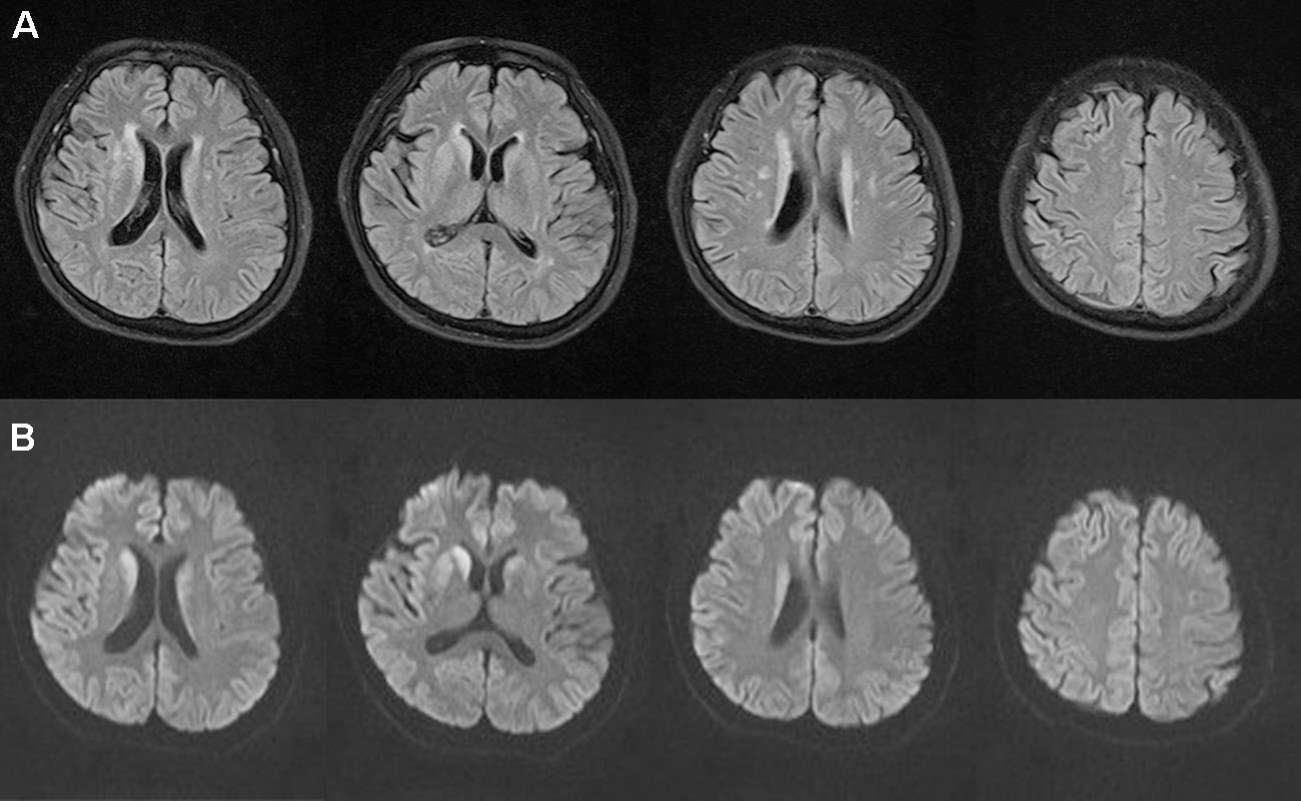
Brain magnetic resonance imaging magnetic. Fluid-attenuated inversion recovery **(A)** showed hyperintensities in the bilateral frontal lobe, corona radiate, and near the anterior and posterior horns of the lateral ventricle. Diffusion-weighted imaging **(B)** showed hyperintensities in the bilateral caudate nucleus and putamen on November 1, 2017.

Ms. S was a 63-year-old female with no previous medical or psychiatric history. In July 2017, she presented with dizziness, weakness, chronic shoulder pain, and high blood pressure. She informed her family that she felt helpless and sick. The preliminary examination revealed nothing but multiple lacunar infarcts in brain magnetic resonance imaging (MRI) scans. On September 17, 2017, she exhibited anhedonia, fear, anxiety, impatience, and a propensity to cry after being annoyed with others. She was examined in the psychiatric unit of the local hospital. Her value on the Self-rating Depression Scale (SDS) was 53.75, which pointed to mild depression, whereas on the Hastgawa Dementia Scale (HDS), she scored 13.0, which suggested probable dementia (education: primary school). The memory quotient (MQ) of Wechsler Memory Scale (WMS) was 59. Her sleep was normal. She was diagnosed with depression, and sertraline 50 mg/day was prescribed. Her symptoms nonetheless worsened with insomnia, garrulity, irritability, and gait imbalance. Her memory function deteriorated, and she became disoriented. The psychiatrist changed the antidepressant drug to venlafaxine 75 mg/day on October 8, 2017. However, instead of improving, the condition rapidly worsened. Her speech became hypophonic and monotonous with a paucity of content. She was sleepy during the day and sometimes burst into tears. Her arms curled up, indicating panic. She developed psychomotor retardation, responded poorly to questions, experienced visual hallucination, and suffered from a rigid posture with paroxysmal myoclonus and an inability to walk. The changes in her symptoms were initially considered to be side effects of venlafaxine. Two weeks later, she had deteriorated further and was unable to talk, exhibiting dysphagia and suffering from urinary incontinence. The symptoms did not improve after the withdrawal of the antidepressant. An assessment of her electroencephalogram (EEG) revealed generalized slow activity (**Figure 1A**). She was then transferred to the neurologic ward of our hospital where the following neurological findings were detected: akinetic mutism (AM), normal muscle strength, increased muscle tension, brisk tendon reflexes, and unresponsive pathologic reflexes. We performed a hematology screen for endocrine, metabolic, autoimmune, neoplastic, and infectious diseases, which were all negative. Cerebrospinal fluid (CSF) studies, including a paraneoplastic, an autoimmune antibody panel, and a tubercular, fungal antibody survey were also negative. Fluid-attenuated inversion recovery (FLAIR) of the brain MRI showed hyperintensities in the bilateral frontal lobe, corona radiate, and near the anterior and posterior horns of the lateral ventricle (**Figure 2A**). Diffusion-weighted imaging (DWI) showed hyperintensities in the bilateral caudate nucleus and putamen (**Figure 2B**). A diagnosis of CJD was considered. One week after admission, the second EEG was performed, revealing partially periodic sharp wave complexes (PSWC; **Figure 1B**). No gene mutations associated with genetic CJD were found, but methionine homozygotes were detected at codon 129 of the prion protein gene. The final diagnosis was probable sCJD according to the diagnostic criteria for sCJD (20, 21). Antibiotics, antiviral, and corticosteroid therapies had been tried since admission, but none of them worked. Ultimately, she was discharged from the hospital.

## Methods

### Search Strategy and Study Selection

We searched PubMed, EMBASE, and PsycINFO up to May 2018 for previous cases using the key words “Creutzfeldt–Jakob disease AND depression.” The reports were restricted to those published and unpublished in English and those including human subjects. The inclusion criteria were as follows: case reports, case series, previous literature reviews, or systematic reviews describing sCJD patients with depression as the first symptom and receiving the treatment for depression. The CJD patients had to meet the WHO or 2009 Consortium diagnostic criteria for definite or probable sCJD (20, 21). To minimize confounders, such as the effect of other medications on outcomes, the included cases were those in which depression was the only symptom diagnosed initially. Two authors independently decided on the selection.

### Data Extraction

The data extracted included study name, study characteristics, patient characteristics, and the duration, institutional care, symptoms, examinations, treatments, and diagnosis of distinct phases. The duration of CJD patients was divided into three phases based on the main symptoms. The first phase was the prodromal phase, with mental manifestations; the second phase was the intermediate phase, with progressive dementia, myoclonus, psychiatric disorder, pyramidal signs, and extrapyramidal performance; the third phase was the late phase, with incontinence, AM, coma, or decorticate rigidity. If an article did not distinguish between the duration of the second and third phases, we utilized a value of half of the total duration of the two periods as their respective durations. Data were graded by two authors independently according to the Oxford Centre for Evidence-Based Medicine levels of evidence (22).

### Statistical Analysis

A systematic analysis was performed. Categorical variables were described using proportions and continuous variables using medians and interquartile range (IQR). A Kaplan–Meier survival analysis was conducted in those patients for whom we had data on the three-phase duration and the use of antidepressants. Antidepressants were categorized into three classes, SSRIs, newer non-SSRI antidepressants (mostly SNRIs), and first-generation antidepressants (mostly TCA) according to the Anatomical Therapeutical Chemical (ATC) classification system (World Health Organization, 1999). The log rank test was used to compare the survival distributions of different groups. Finally, a multivariate Cox regression analysis with Enter was undertaken to determine the predictors of survival. Due to the small number of cases, we considered only three factors: gender, age, and antidepressant type. The model with a significant score test and a smaller deviance in likelihood ratio test will be preferred. Significance was set at *P* < .05 (two-sided test). Statistical analysis was completed using SPSS v17.0 (SPSS Inc., Chicago, IL, USA).

## Results

### Study Identification and Characteristics

The Preferred Reporting Items for Systematic Reviews and Meta-Analyses (PRISMA) guidelines were followed (23). The PRISMA flow diagram is depicted in **eFigure** in the **Supplementary Material**. In our literature search, we identified 13 cases from 12 articles that met our inclusion criteria for qualitative analysis (24–35). Subsequently, 11 cases from 10 articles were included for qualitative analysis (24–26, 28, 30–35). With the addition of our case, a total of 12 subjects could be included in the Kaplan–Meier survival and multivariate Cox regression analysis.

The characteristics and evidence levels of the 14 cases published from 1993 to 2017 are shown in **eTable** in the **Supplementary Material**. All included articles were case reports. The age of all subjects was 58.8 (55.5–61.5) years with 11 (79%) being female. After administration of antidepressants, only 1 case out of 13 (8%) showed improved depressive symptoms.

### Survival Time of Sporadic Creutzfeldt–Jakob Disease Patients with Different Antidepressants

**Figure 3:**
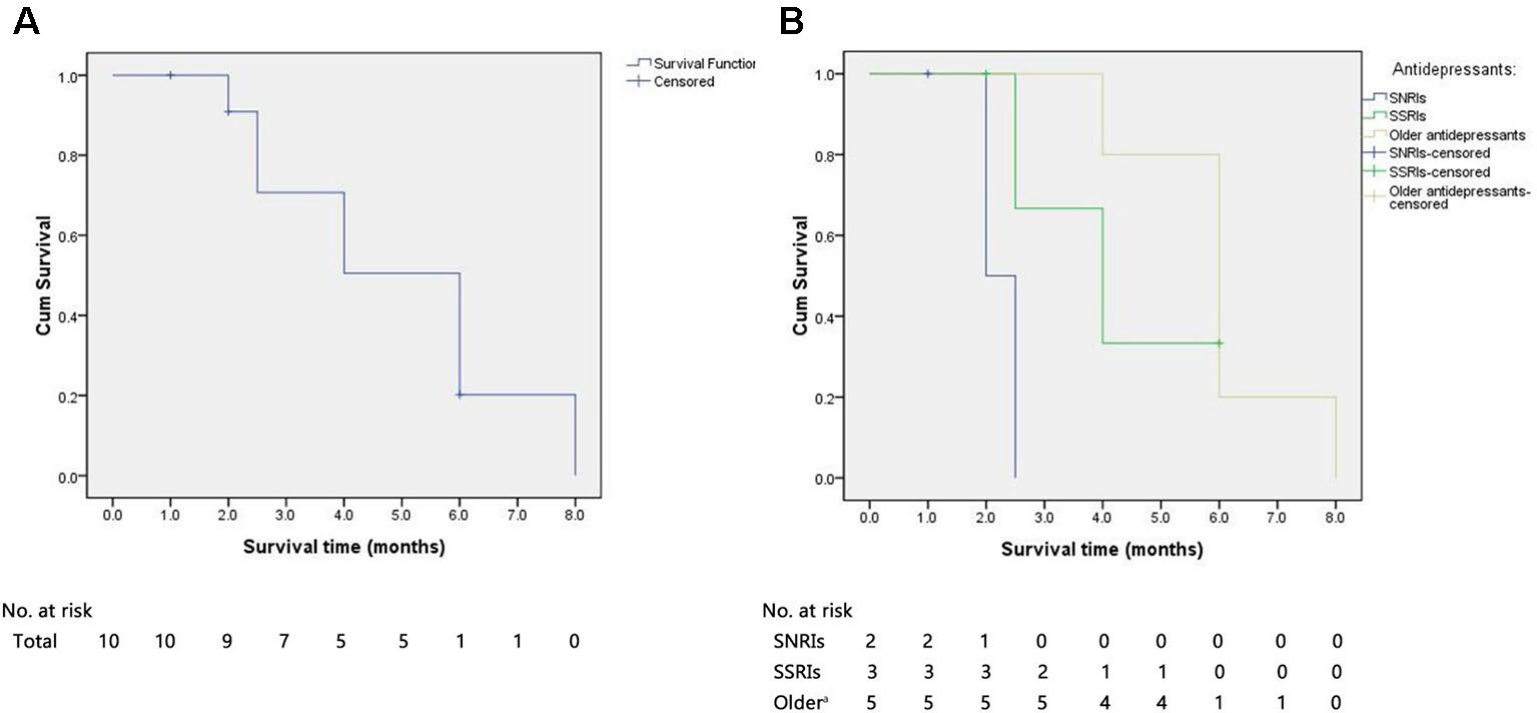
The Kaplan–Meier survival curves for sCJD patients with antidepressants. **(A)** Survival time for all patients. **(B)** Survival time for patients stratified according to the type of antidepressant. X-axis represents survival time (months) and Y-axis represents survival probability. sCJD = sporadic Creutzfeldt–Jakob disease; SNRIs = serotonin and noradrenaline reuptake inhibitors; SSRIs = selective serotonin reuptake inhibitors. ^a^Referred to first-generation antidepressants.

A Kaplan–Meier survival curve for all of the sCJD patients who had used antidepressants is shown in **Figure 3**. The median survival time of all of the cases was 6.0 months. The cumulative incidences with survival times less than 3, 6, and 12 months were 30.0%, 90.0%, and 100%, respectively. All of the patients died within 1 year after onset.

The use of antidepressants in 12 cases is as follows: 3 (25%) were given SNRIs (1 censored), 4 (33%) were administered SSRIs (2 censored), and 5 (42%) were treated with first-generation antidepressants. The median survival times for cases with SNRIs, SSRIs, and first-generation antidepressants were 2.0, 4.0, and 6.0 months, respectively. The median survival time of patients with SNRIs was significantly shorter than those treated with first-generation antidepressants (log rank, *P* = .008) and relatively shorter than those with SSRIs (log rank, *P* = .090). Furthermore, the median survival time of patients receiving SSRIs was nonsignificantly shorter than those with the first-generation antidepressants (log rank, *P* = .615).

### Predictors of Survival Time in Sporadic Creutzfeldt–Jakob Disease Patients With Depression

The Cox regression model including age and antidepressant types (**Table 1**) was preferred (likelihood ratio test, deviance = 25.469; score test, *P* = .043). Compared to first-generation antidepressants, the use of SNRIs [hazard ratio (HR), 23.028; 95% confidence interval (CI), 1.401 to 378.461; *P* = .028] remained independently associated with significantly shorter survival time in sCJD patients with depression.

**Table 1:** Cox regression analysis of survival time in sCJD patients with depression.

Factors	Unadjusted		Adjusted*^a^*	
	HR (95% CI)	*P*	HR (95% CI)	*P*
Age	1.015 (0.947–1.087)	.677	1.042 (0.955–1.136)	.354
Antidepressants	NA	.103	NA	.086
SNRIs	15.665 (1.215–201.884)	.035*	23.028 (1.401–378.461)	.028*
SSRIs	1.432 (0.256–8.018)	.683	2.689 (0.295–24.498)	.380
First-generation antidepressants (reference)	NA	NA	NA	NA

sCJD, sporadic Creutzfeldt–Jakob disease; HR, hazard ratio; C, confidence interval; NA, not applicable (outcome not investigated); SNRIs, serotonin and noradrenaline reuptake inhibitors; SSRIs, selective serotonin reuptake inhibitors.

a Adjusted for age and antidepressant types.

*P < .05.

## Discussion

Since depression is one of the most common global mental health conditions, the use of antidepressant drugs has increased dramatically with almost half of the prescriptions being for some off-label indication (36). However, our investigation revealed that almost none of the sCJD patients experienced any relief of their depressive symptoms after the antidepressant treatment. Furthermore, the median survival time of sCJD patients receiving SNRI therapy was shorter than the average survival of sCJD patients (2.3 vs. 4.6–17.4 months) (16). Thus, antidepressants do not seem to have any beneficial effect on sCJD patients with depression, a finding consistent with previous clinical studies not only on sCJD patients but also those with dementia (10, 19). Likewise, the efficacy characteristics of antidepressants indicate that antidepressants appear to display relatively poor efficacy in people older than 65 years (37). Based on the neurotransmitter receptor hypothesis of antidepressant drugs, the amount of neurotransmitter changes rapidly after an antidepressant is administered. But the clinical effects appear only weeks later (usually 6–12 weeks) (37). Due to the rapid progress of the disease, sCJD patients often use antidepressants for only a brief period. Therefore, the drugs usually cannot achieve clinical efficacy but are instead likely to exert unwanted side effects.

The question arises as to why patients with sCJD receiving antidepressants seem to deteriorate faster. Since antidepressants mainly alter neurotransmitter levels, we postulate that this deterioration must be related to these changes. Several independent lines of evidence support this postulation. sCJD resembles the degenerative dementias. Studies of AD, the best-known of the degenerative dementias, have proved that the accumulations of β-amyloid (Aβ) and tau proteins damage neurons and synapses, whereas the change in neurotransmitters such as acetylcholine (ACh) occurs at the initial stage (38). Similarly, the cause of sCJD neuropathological changes has also proved to be a reversible process, such as synaptic or neuronal dysfunction (39). Interestingly, patients with sCJD also have higher concentrations of Aβ and tau proteins in their serum and CSF (40, 41). Aβ may be propagated in a prion-like manner (42, 43). Similar observations have been made for tau (44). Because sCJD and AD share these common features (45), perhaps we can also attempt to delay the progress of sCJD by regulating the level of neurotransmitters. Furthermore, the typical lesions in MRI and histologic appearance in sCJD consist of cortical, basal ganglia, and cerebellum (46, 47). It was observed that the clinical target areas in the brainstem of prion-infected mice were the locus coeruleus, the nucleus of the solitary tract, and the pre-Bötzinger complex (48). These brain areas are exactly those in which the neuronal cell bodies generating neurotransmitters are mainly located or the areas innervated by their axonal projections.

How do these neurotransmitters modulate disease progression? According to our study, the survival period of sCJD patients is related to the type of antidepressants. By analyzing the pharmacological characteristics, we postulate that elevations in 5-hydroxytryptamine (5-HT) and norepinephrine (NE) may worsen the condition, although the sedative effects mediated by anti-histamine (HA), anti-ACh, and blockade of α-1 adrenoceptors may contribute to the relief of symptoms. Acute stimulation of the 5-HT can produce symptoms similar to sCJD (37). Neurotransmitters exist in many brain areas, but which area plays the key role? When SSRI treatment is initiated, the concentrations of 5-HT are elevated to a much greater extent at the somatodendritic area located in the midbrain raphe, rather than in the brain areas where the axons terminate (37). Therefore, SSRIs may exert more significant effects on the brainstem in patients with sCJD. However, the pathological changes in sCJD do not occur in the brainstem but rather in the projection pathways of neurotransmitters, such as cortical, basal ganglia, and cerebellum. This raises the question of how changes in the brainstem’s neurotransmitter activities affect other brain areas. Taking into account the symptoms (such as myoclonus that occurs at night, AM) of patients with sCJD, we postulate that one pathway through which brainstem’s neurotransmitter activities trigger cognitive impairment encountered in sCJD patients may be through its disruption of sleep centers in the brainstem. AM is a disorder caused by damage to the ascending reticular activating system (ARAS) centered on the brainstem. Arousal is regulated by ARAS, which is influenced in large part by five key neurotransmitters: HA, dopamine (DA), NE, 5-HT, and ACh. Changes in these neurotransmitters can cause sleep disorders, i.e., rapid eye movement (REM) sleep without atonia (RSWA), and nonrapid eye movement (NREM) sleep disruption. Clinical studies have shown that SSRIs and SNRIs are associated with a higher prevalence of RSWA (49), explained in part by the theories about REM sleep initiation that advocate for a double switch model, possibly triggered by neurons located in the brainstem (50). Sleep disorders can cause many symptoms similar to sCJD, such as psychiatric symptoms (fear, anger, aggressive behavior, etc.), increased muscle tone, and most notably, cognitive impairment. For example, REM sleep behavior disorder (RBD) is by far the strongest clinical predictor of onset of neurodegenerative diseases (51). The presence of RBD in Parkinson’s disease (PD) is associated with higher risk of cognitive decline (52). The reduced NREM slow-wave activity (SWA) generation was associated with impaired hippocampus-dependent memory consolidation (53). The Aβ burden in the medial prefrontal cortex correlates significantly with the severity of impairment in NREM SWA generation (53). Even one night of sleep deprivation could result in a significant increase in Aβ burden in the brain (54). Thus, the dual excitatory effects of 5-HT and NE may exacerbate the sleep deprivation encountered in sCJD patients, causing a cascading effect and then triggering cognitive impairment.

Why is the effect of neurotransmitters so rapid in sCJD patients? One reason may be the pathological overactivity of the brain’s serotonergic system in this disease. This hypothesis is supported by the evidence that the mean tryptophan hydroxylase (TPH)-positive cell size was significantly greater and cells were more intensely stained in CJD compared to controls (55). This may result in an increase in release of 5-HT. Coupled with the cascade effect of neurotransmitters, the actual effects may be amplified. The increase in 5-HT also reduced the release of DA in the prefrontal zone by negative feedback regulation (37). The reduction of DA may cause some symptoms similar to sCJD, such as cognitive impairment and apathy. Another reason could be that synapses in sCJD may be more vulnerable. The pathological features of CJD indicate that the vacuole in the cytoplasm is the cystic dilation of neurons and necrosis of the necrotic membrane. The cell membrane damage of CJD seems to be more serious than AD, where amyloid plaques form outside the neurons and neuron fibers entangle within the neurons. Damage to the synaptic membrane leads to a decrease in neurotransmitter receptors. In response to this change, the remaining receptors may be in a hypersensitivity state, or the number of receptors may increase (37), which may further enhance the effects of neurotransmitters such as 5-HT.

Many of the families of patients with sCJD complain of the delay in diagnosis and the plethora of misdiagnoses (56). Expediating a sCJD diagnosis is of great significance. Almost half of patients were misdiagnosed first as “psychiatric patients” (57). Consequently, it is very important for psychiatrists to consider CJD among the possible differential diagnoses in elderly patients. Our investigation suggests that it may be helpful to use imaging such as functional MRI and positron emission computed tomography (PET) to detect earlier changes in patients with sCJD.

Of course, our study has some limitations. First, the number of available cases is too small, and in many cases, the description of psychiatric symptoms and details of the antidepressants were inadequate. Second, because case reports tend to report exceptional situations, there is inevitably some bias. However, we think it is a reasonable approach to study CJD by undertaking case analysis or studies of one single individual. Due to the rapid progression of CJD, studies on population samples often overlook certain unique changes.

Investigations into sCJD have mainly focused on autopsy-based pathology, but little is known about neurophysiological changes. We hope this study will draw attention to the depressive symptoms of sCJD patients and the underlying neurophysiological mechanisms.

## Conclusions

The use of antidepressants was associated with a shorter survival time of sCJD patients, especially the use of SNRIs. The possible neurotransmitter changes may be due to a pathophysiological mechanism in CJD. Functional imaging and use of the polysomnogram to detect earlier changes in sCJD patients may be worth trying.

## Ethics Statement

This case study was carried out in accordance with the recommendations of the Ethical Committee of China Medical University. The case study has been approved by the Ethics Committee of China Medical University. The subject gave written informed consent in accordance with the Declaration of Helsinki. Written informed consent was also obtained from each patient for the publication of this case report.

## Author Contributions

CZ had full access to all of the data in the study and takes responsibility for the integrity of the data and the accuracy of the data analysis. CZ and YLia contributed to the study concept and design. YLi contributed to the case report. YLia, YLi, MG, and SZ contributed to the acquisition, analysis, or interpretation of data. YLia, HW, and XC drafted the manuscript. CZ conducted the critical revision of the manuscript for important intellectual content. All authors performed the statistical analysis.

## Funding

This work was supported by The Liaoning Province Key Research and Development Project Critical Project (no. 2017225005, CZ), The Shenyang Municipal Bureau of Science and Technology International Exchange and Cooperation Project (no. 17-129-6-00, CZ), and China Medical University High-level Innovation Team Training Plan (no. 2017CXTD02, CZ).

## Conflict of Interest Statements

The authors declare that the research was conducted in the absence of any commercial or financial relationships that could be construed as a potential conflict of interest.
