# Supplementary figures and images for: Does the Use of Antidepressants Accelerate the Disease Progress in Creutzfeldt–Jakob Disease Patients With Depression? A Case Report and A Systematic Review
Source: Front Psychiatry. 2019 May 3;10:297. doi: 10.3389/fpsyt.2019.00297 (PMC6509196; doi:10.3389/fpsyt.2019.00297)

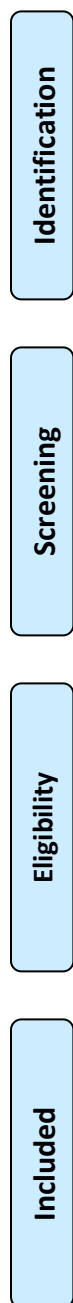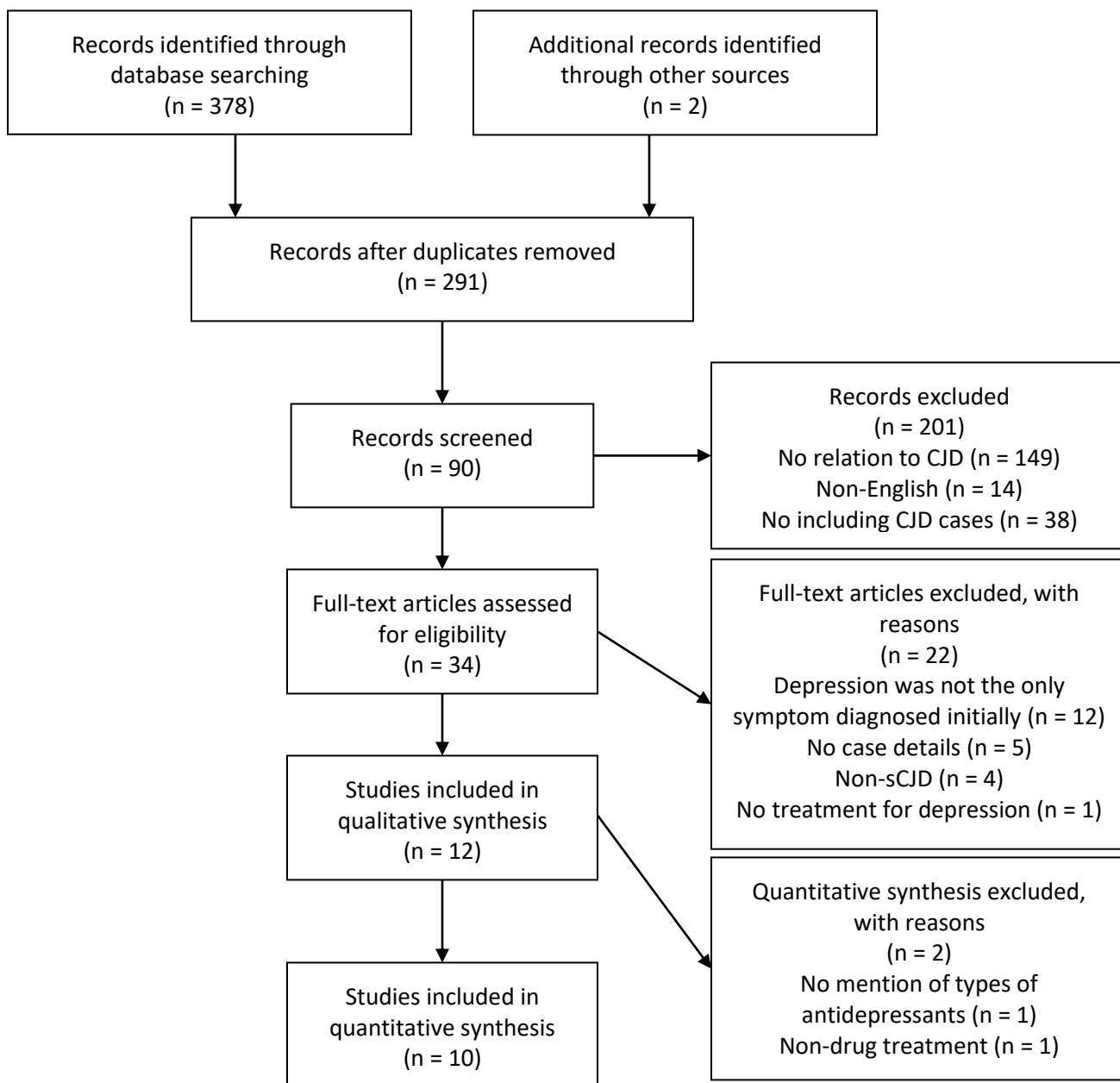

Supplement: Supplementary file 1 [file Image_1.pdf]
