# Supplementary material for: Does the Use of Antidepressants Accelerate the Disease Progress in Creutzfeldt–Jakob Disease Patients With Depression? A Case Report and A Systematic Review
Source: Front Psychiatry. 2019 May 3;10:297. doi: 10.3389/fpsyt.2019.00297 (PMC6509196; doi:10.3389/fpsyt.2019.00297)
Supplement: Supplementary file 2 [file Table_1.pdf]

eTable. The characteristics and evidence levels of included cases about sCJD patients with depression

Source	Evidence level	Sex	Age	Phase[a]	Duration	Institution[b]	Symptoms	EEG[c]	MRI[d]	CSF[e]	Gene[f]	Biopsy[g]	Treatments[h]	Diagnosis
Liang, 2017	5	F	63	1	3 m	P	Depressive disorder, confusion, cerebellar signs.						Sertraline, and then venlafaxine	Depression
				2	2 w	P	Extra pyramidal signs, myoclonus, visual signs.							Stupor
				3	> 2 w	N	Akinetic mutism.	+	+		MM			Probable sCJD
Wang, 2017[35]	5	M	75	1	4 m	GP	Depressive disorder.						Sertraline	Catatonic depression
				2	60 d	P	Extra pyramidal signs.	+	+	+				Probable sCJD
				3	Alive	C	NR							Probable sCJD
Milanlioglu, 2015[34]	5	F	51	1	2.5 m	P	Extra pyramidal signs, memory difficulties.							Catatonic depression
				2	29 d	N	Myoclonus.	+	+	+			Trazodone	Probable sCJD
				3	29 d	N	Akinetic mutism.							Probable sCJD
Yang, 2013[33]	5	M	57	1	7 m	P	Depression.						Sertraline[i]	Major depressive disorder
				2	2 w	N	Myoclonus, extra pyramidal signs, incontinence		+					Probable sCJD
				3	Months	N+C	Akinetic mutism.	+		+	MM			Probable sCJD
Power, 2012[32]	5	F	59	1	3 m	GP	Irritability, suspiciousness.						Sertraline	Depression
				2	Weeks	P	Cerebellar signs, myoclonus, incontinence.	+	+	+				Probable sCJD
				3	4 m	C	Inappropriate behaviors.							Probable sCJD
González-Duarte, 2011[30]	5	F	38	1	5 m	NR	Depressive disorder.						SSRIs	Mood depression
				2	5 m	NR	Visual signs, extra pyramidal signs, cerebellar signs.	+	+	+				Probable sCJD
				3	> 1 m	NR	Akinetic mutism.							Probable sCJD

Source	Evidence level	Sex	Age	Phase[a]	Duration	Institution[b]	Symptoms	EEG[c]	MRI[d]	CSF[e]	Gene[f]	Biopsy[g]	Treatments[h]	Diagnosis
Grande, 2011[31]	5	F	80	1	3 m	P	Mood disorder with psychotic symptoms.						Different antidepressants	Catatonic depression
				2	25 d	P	Extra pyramidal signs, poor mental state.	+		+	MV		ECT	Probable sCJD
				3	25 m	N	Akinetic mutism.					+		sCJD
Muayqil, 2007[29]	5	M	58	1	15 m	NR	Depressive disorder, poor mental state.						Venlafaxine	Depression
				2	2 m	NR	Cerebellar signs.	+						Probable sCJD
				3	2 w	NR	Dementia, myoclonus.		+			+		sCJD
Jardri, 2006[28]	5	F	50	1	6 m	P	Major depressive disorder with psychotic symptoms.						Paroxetine + mianserine, and then amitriptyline	Major depressive disorder
				2	3 m	N	Myoclonus, cerebellar signs, extra pyramidal signs.		+	+			Amitriptyline	Probable sCJD
				3	3 m	N	Akinetic mutism.	+						Probable sCJD
Jiang, 1999[27]	5	F	68	1	6 w	P	Depressed mood.						ECT	Major depression
				2	NR	N	Dementia, myoclonus.	+		+		+		sCJD
				3	NR	C	Akinetic mutism, decorticate posturing.							sCJD
Goetz, 1993[25]	5	F	73	1	9 m	NR	Depression, confusion.						Doxepin	Depressive pseudodementia
				2	4 m	NR	NR						ECT[i] + Nortriptyline	Probable sCJD
				3	2 m	NR	Confusion, myoclonus, extra pyramidal signs.	+				+		sCJD
Onofrj, 1993[26]	5	F	61	1	2 m	P	Depression.						TCA	Depression
				2	4 m	N	Cerebellar signs, extra pyramidal signs, myoclonus.		+					Probable sCJD
				3	2 m	NR	Incontinent.	+				+		sCJD

Source	Evidence level	Sex	Age	Phase[a]	Duration	Institution[b]	Symptoms	EEG[c]	MRI[d]	CSF[e]	Gene[f]	Biopsy[g]	Treatments[h]	Diagnosis
Azorin, 1993[24]	5	F	57	1	14 m	N	Mood disorder, memory difficulties, visual signs.						Amitriptyline	Pseudodemential depression
				2	2 m	P	Auditory hallucinations, pyramidal signs, myoclonus.							Probable CJD
				3	2 m	N	NR	+				+		CJD
Azorin, 1993[24]	5	F	59	1	NR	NR	Mood disorder, language difficulties.						Viloxazine	Pseudodemential depression
				2	2 m	NR	NR	+						Probable CJD
				3	6 m	NR	NR					+		CJD

[a] The duration of CJD was divided into three phases based on the main symptoms: 1 the prodromal phase, 2 the intermediate phase, 3 the late phase.
[b] The institution was divided into four classes: psychiatric department (P), neurologic department (N), General Practitioner (GP), care nursing center (C).
[c] + represents a typical electroencephalograms (EEG) (periodic sharp curve complexes).
[d] + represents magnetic resonance imaging (MRI) high signal abnormalities in caudate nucleus and/or putamen or diffusion-weighted imaging or fluid attenuated inversion recovery (FLAIR).
[e] + represents a positive 14-3-3 cerebrospinal fluid (CSF) assay.
[f] The genotype at codon 129 of the prion protein gene includes methionine homozygotes (MM), methionine/valine heterozygotes (MV) and so on.
[g] + represents a typical biopsy (cavernous degeneration).
[h] Only includes treatments for depression.
[i] The patient had improved depressive symptoms after the treatment.
F = female; M = male; sCJD = sporadic Creutzfeldt–Jakob disease; SSRIs = selective serotonin reuptake inhibitors; TCA = tricyclic antidepressants; ECT = electroconvulsive therapy; NR = not reported.
